# Circulating monocytes accelerate acute liver failure by IL‐6 secretion in monkey

**DOI:** 10.1111/jcmm.13673

**Published:** 2018-07-11

**Authors:** Gang Guo, Yongjie Zhu, Zhenru Wu, Hongjie Ji, Xufeng Lu, Yongjie Zhou, Yuanmin Li, Xiaoyue Cao, Yanrong Lu, Prue Talbot, Jiayu Liao, Yujun Shi, Hong Bu

**Affiliations:** ^1^ Laboratory of Pathology Key Laboratory of Transplant Engineering and Immunology NHFPC West China Hospital Sichuan University Chengdu China; ^2^ Department of Cell Biology & Neuroscience University of California Riverside CA USA; ^3^ The UCR Stem Cell Center and Core University of California Riverside CA USA; ^4^ Department of Bioengineering University of California Riverside CA USA

**Keywords:** acute liver failure, interleukin‐6, monocyte, non‐human primate

## Abstract

Acute liver failure (ALF) is associated with high mortality, and a poor understanding of the underlying pathophysiology has resulted in a lack of effective treatments so far. Here, using an amatoxin‐induced rhesus monkey model of ALF, we panoramically revealed the cellular and molecular events that lead to the development of ALF. The challenged monkeys with toxins underwent a typical course of ALF including severe hepatic injury, systemic inflammation and eventual death. Adaptive immune was not noticeably disturbed throughout the progress of ALF. A systematic examination of serum factors and cytokines revealed that IL‐6 increase was the most rapid and drastic. Interestingly, we found that IL‐6 was mainly produced by circulating monocytes. Furthermore, ablation of monocyte‐derived IL‐6 in mice decreased liver injury and systemic inflammation following chemical injection. Our findings reveal a critical role of circulating monocytes in initiating and accelerating ALF, indicating a potential therapeutic target in clinical treatment for ALF.

## 1 INTRODUCTION

Acute liver failure (ALF) is a complicated clinical syndrome that consists of metabolic and immunological dysfunction, hepatic encephalopathy, coagulopathy, sepsis and, eventually, multi‐organ failure. ALF is caused by sudden and severe hepatic injury, and because of the high morbidity and mortality rate, ALF is the most common cause for emergency liver transplantation.^1^, ^2^, ^3^, ^4^, ^5^ However, the shortage of transplantable donor livers and lifelong immunosuppression has restricted the use of this intervention, making it necessary to develop new therapeutic methods.

The pathophysiological process underlying ALF varies based on its cause and is still poorly understood.^2^, ^3^, ^6^ Immune system disarrangement, especially the dysregulation of the innate immune system, has been regarded as the core event in this process. Hepatocyte death leads to the release of damage‐associated molecular patterns (DAMPs), which trigger the innate immune response and the production of pro‐inflammatory mediators such as TNFα, IL‐1 and IL‐6 and reactive nitrogen and oxygen species.^7^ Within the innate immune system, hepatic resident macrophages known as Kupffer cells (KCs) are critical for initiating the transduction and amplification of “alarm” signals following hepatocyte injury. The expansion of hepatic macrophages, which derived from bone marrow‐derived precursors—circulating monocytes (c‐Mos)^8^, ^9^, ^10^— leads to the systemic release of cytokines, growth factors and chemokines to recruit effector cells (eg neutrophils) to restore homeostasis. However, under certain conditions, uncontrolled hypercytokinemia, also described as a “cytokine storm,” exacerbates tissue destruction and accelerates systemic inflammatory response syndrome (SIRS).^7^, ^10^, ^11^ In humans, ALF‐associated mortality is often attributable to severe SIRS and the complications associated with recurrent sepsis and multi‐organ dysfunction.^1^, ^3^, ^9^ Depleting activated macrophages and plasmapheresis that alleviates hypercytokinemia has been shown a promise as a treatment for ALF,^8^, ^12^ but has not yet reached the clinical stage.

The lack of the precise cellular and molecular bases of ALF has largely hindered the development of therapeutic strategies. The current knowledge of the pathophysiological processes underlying ALF comes from small animal studies and is limited by substantial differences in species.^13^, ^14^, ^15^ In addition, their blood and tissue volumes are limited. Small animals cannot be used to obtain consecutive samples for examination, thus making it infeasible to generate continuous dynamic data from an individual animal. Human patients with ALF are hospitalized when symptoms appear, and any information related to the latent stage of this disease is already too late to obtain.^1^, ^3^, ^16^ Moreover, a high risk of bleeding and low compliance to biopsies render analyses of progressive histological changes impossible.

A satisfactory animal model of ALF should meet several criteria, including reversibility, reproducibility, death from liver failure, the presence of a therapeutic window and adequate animal size.^13^, ^14^ Moreover, it is important to select a species with metabolic and physiological properties similar to those of humans. We previously established a non‐human primate (Macaca mulatta) model of ALF, in which we induced ALF with a single intraperitoneal injection of low‐dose α‐amatoxin and lipopolysaccharide (LPS).^17^ Amatoxin is an alkaloid derived from poisonous mushrooms that selectively inhibits RNA polymerase II.^18^ When taken up by hepatocytes, amatoxin is released in its original form in the bile and then appears in the enterohepatic circulation. Before it is fully cleared within 24 hours, amatoxin repeatedly damages hepatocytes without causing clear injury to extrahepatic organs. Acute liver injury is often augmented by gut‐derived endotoxins, and LPS alone or in combination with other agents is widely used to establish animal models of ALF.^18^, ^19^ Following the injection of toxins, the monkeys displayed changes in clinical features, hepatic indexes, histopathology, imaging and lifespan that were typical of the progress observed in the clinical ALF.^17^ These data indicate that our animal model is appropriate for exploring the pathophysiology of ALF and for evaluating potential therapeutic strategies.

Here, using a toxin‐induced monkey model of ALF, we investigated the molecular and cellular mechanism that initiates and promotes ALF. We reported that the early excessive activation of circulating monocytes, rather than the resident Kupffer cells, plays a critical role in the augmentation of systemic inflammation, which act as a potential therapeutic target for this lethal syndrome.

## 2 MATERIALS AND METHODS

### 2.1 Animals

**Figure 1:**
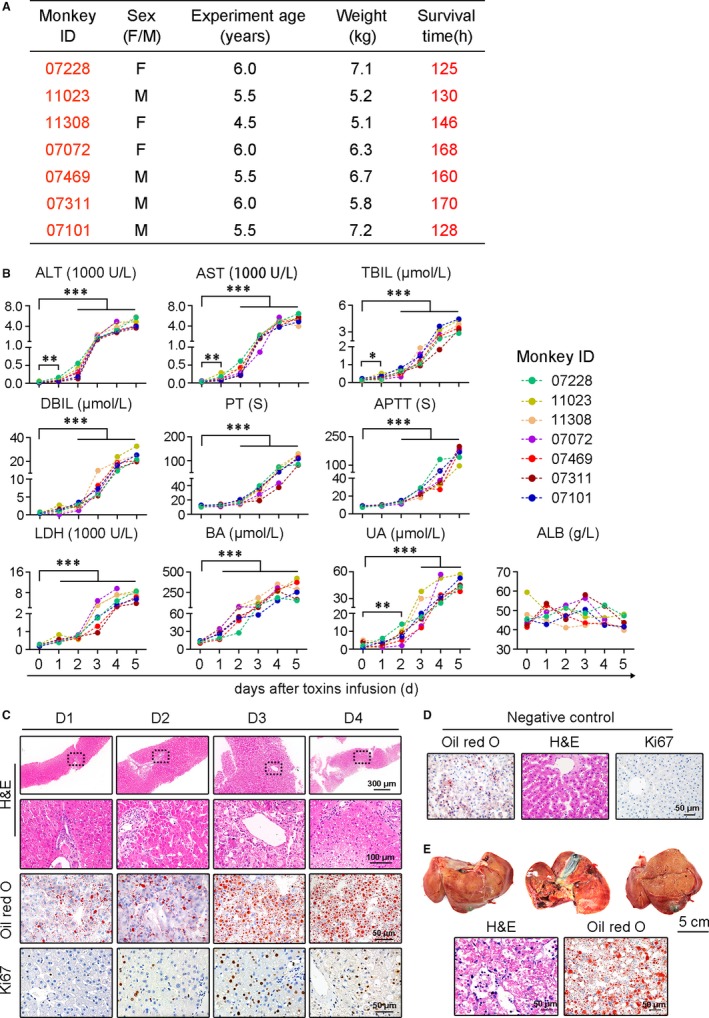
Monkeys develop lethal acute liver failure following toxin treatment. A, The detail information and survival time of each monkey. B, Biochemical assays of hepatic indexes. (ALB, albumin; ALT, alanine aminotransferase; APTT, activated partial thromboplastin time; AST, glutamicoxaloacetic transaminase; BA, blood ammonia; DBIL, direct bilirubin; LDH, lactate dehydrogenase; PT, prothrombin time; TBIL, total bilirubin; UA, uric acid). *P < .05, **P < .01, ***P < .001 compared with the data at 0 d. C, HE staining, Oil red O staining and Ki67 immunohistochemical staining of liver specimens at the indicated times. D, HE staining, Oil red O staining and Ki67 immunohistochemical staining of healthy monkey liver. E, Gross and histopathological changes in a failed liver

The animal protocols used in this study were approved by the Institutional Animal Care and Use Committee of the Traditional Chinese Medicine National Center (Chengdu, China; Protocol: IACUC‐2012001C). Adult healthy experimental rhesus monkeys were provided by Chengdu Ping'an Experimental Animal Reproduction Center (License No.: SCXK [CHUAN] 2014‐013, Chengdu, China). For detailed information regarding the monkeys, see Figure 1A.

The monkeys were housed singly in standard cages with a 12 hours light/dark cycle. For all invasive operations, the monkeys were anaesthetized using ketamine and propofol to subsequent intubation and ventilation using isoflurane to maintain anaesthesia. To establish a toxin‐induced model of ALF, the monkeys were intraperitoneally administered α‐amatoxin (Alexis Biochemicals, Lausen, Switzerland) at 25 μg/kg bodyweight (BW) and LPS (Sigma‐Aldrich, St. Louis, MO) at 1 μg/kg BW as described previously, with minor modifications.^1^ The α‐amatoxin and LPS were diluted in 50 mL of physiological saline and slowly infused into the peritoneal cavity. All monkeys were provided with standard dry monkey food and water ad libitum and supported with antibiotics, glucose, branched chain amino acid and fat milk injections following the injection of the toxins. Blood samples were collected and core needle liver biopsies were guided by ultrasonography scans at indicated time‐points after the administration of the toxins. The state of consciousness and behaviour of the animals was monitored, and hepatic encephalopathy was defined according to the West Haven criteria used in humans. The monkeys were scheduled for euthanasia when they displayed the following symptoms: disable to eat and drink, flapping tremor, a hepatic coma‐like state and unable to wake up. For euthanasia, the monkeys were intramuscularly injected with 15 mg/kg BW ketamine as the basal anaesthesia; they were then bled to death after a sufficient depth of anaesthesia was induced by intravenous injection with 100 mg/kg BW of pentobarbital sodium. After euthanasia, the monkeys were submitted to a full necropsy.

### 2.2 Histology

The monkey liver specimens were subjected to frozen section for oil red O staining and fluorescence immunohistochemistry. For H&E staining, tissues were fixed in 10% neutral buffered formalin for 48 hours and embedded in paraffin, sectioned at 5 μm and staining with hematoxylin and eosin. Immunohistochemistry and immunofluorescence of Ki67 antibody (1:100, Thermo Fisher, Grand Island, NY) were used to measure cell proliferation. CD68 (working solution, Abcam, Cambridge, UK) and MAC387 (1:200, Thermo Fisher, Grand Island, NY) were used to mark the resident hepatic macrophages and recruited circulating monocytes, respectively.

### 2.3 Biochemical evaluation

Blood serum and plasma was isolated by centrifugation at 1500 *g* for 10 minutes at 4°C for biochemical evaluation and cytokine quantification. All the hepatic parameters were analysed in a standard clinical laboratory in West China Hospital, Sichuan University.

### 2.4 Monkey cytokine level analysis

A Cytokine Monkey Magnetic 29‐Plex Panel (Life technologies, Camarillo, CA) was used to quantify the levels of monkey serum cytokines, chemokines and growth factors on a Luminex 200 System (Millipore, Billerica, MA, USA) according to the manufacturer's instructions.

### 2.5 Assays of sCD163 in monkey serum

Circulating soluble CD163 (sCD163) was assayed by Human CD163 Quantikine ELISA Kit (R&D Systems, Minneapolis, MN). The levels of sCD163 were examined according to the instruction for quantitative detection after serum samples were diluted 100 times. Samples were drawn in 9 mL EDTA (ethylenediaminetetraacetic acid) tubes. The tubes were centrifuged at 4°C for 10 minutes at 1500 g. The plasma was then removed, aliquoted and stored at −80°C until analysis. The levels of sCD163 were analysed in duplicate using frozen serum samples with an in‐house sandwich ELISA on a BEP2000 ELISA analyzer (Dade Behring, Marburg, Germany). The inter‐assay imprecision in this study for sCD163 was 2.4 CV% and 6.2 CV% at levels of 1.97 mg/L and 3.91 mg/L, respectively.

### 2.6 Monkey monocyte isolation

For the isolation of monocytes, the monkeys were instructed to fast overnight and 10 mL of blood was drawn into tubes containing 3 mmol/L EDTA. The buffy coat was isolated by centrifugation (1500 g for 30 minutes at 4°C), and 7 mL of the buffy coat and adjacent plasma was carefully layered onto 3 mL of Histopaque, 1.077 g/mL (Sigma, St. Louis, MO). The leucocytes were separated by centrifugation (400 g for 30 minutes at 25°C). To further purify the monocytes and to eliminate platelets, the cells were washed twice with phosphate buffered saline (PBS) containing 0.1% bovine serum albumin (BSA) and 0.02% EDTA. The cells were recovered by centrifugation (400 g for 15 minutes at 4°C), plated for 2 hours at 37°C in RPMI‐1640 medium, and the adhering cells were harvested for RNA preparation. The purity of adherent monocytes was >90% estimated by flow cytometry using anti CD14 antibody. (BD Biosciences, San Jose, CA). Cell viability of >90% was confirmed using trypan blue exclusion staining.

### 2.7 Circulating lymphocytes isolation

Peripheral blood mononuclear cells (PBMC) from monkeys were isolated by standard density gradient centrifugation (Ficoll‐Hypaque, Sigma Chemicals^®^). Then T cells were harvested using MojoSort™ Human CD3 T cell isolation kit (Biolegend) and B cells were isolated using EasySep™ Human B Cells Enrichment Kit (Stem Cell Technology).

### 2.8 Flow cytometric assay

Assessment of the status of adaptive immune system was performed using monoclonal antibodies against CD3, CD4, CD8 (BD Biosciences, San Jose, CA) and Human Regulatory T Cell (CD4, CD25, FOXP3) Staining Kit (eBioscience, San Diego, CA) and CD1a, CD80, CD86 (eBiosciences, San Diego, CA). Monoclonal antibodies against CD14, CD16 and CCR2 (BD Biosciences) were used to determine monocyte subsets and CCR2 expression. Appropriate negative controls were isotype‐matched mouse MoAbs. Flow cytometry was performed on a Beckman Coulter FC500 and analysed using a Kaluza v1.20 software (Beckman Coulter, Fullerton, CA) or CXP analysis software. On average, 20 000 monocyte‐gated events were acquired.

### 2.9 mRNA isolation and real‐time PCR

Total mRNA was purified from liver tissue homogenates, circulating monocytes and circulating lymphocytes (T and B cells) with the RNeasy Mini kit (QIAGEN, Germany). mRNA was reverse transcribed to cDNA using an iScript cDNA Synthesis kit (Bio‐Rad, Hercules, CA). A CFX Connect Real‐Time System (Bio‐Rad) was used for real‐time PCR. cDNA template was amplified using Sso Advanced Universal SYBR Green Supermix (Bio‐Rad) under standard conditions. Gene expression levels were normalized to *GAPDH* using the comparative CT method. The sequences of IL‐6 gene primers were upstream, 5′‐ATG AGG ACA CTT GCC TGG TG‐3′ and downstream, 5′‐GCT GGC ATT TGT GGT TGG TT. The sequences of *Gapdh* gene primers were upstream, 5′‐TCG AGA GTC AGC CGC ATT TTC‐3′ and downstream, 5′‐GGA ACT TGC CAT GGG TGG AA‐3′.

### 2.10 Fluorescent in situ hybridization (FISH)

For FISH, 2‐mm sections from each paraffin block were prepared. Deparaffinization, pretreatment and protease digestion procedures were performed following the Ribo Fluorescent In Situ Hybridization Kit protocol (Ribobio, Guangzhou, China). IL‐6 mRNA FISH Probe (Red), synthesized by Ribobio (Guangzhou, China), was hybridized at 40°C for overnight. The next day, slides were rinsed and then counterstained with 4′,6‐diamidino‐2‐phenylindole (DAPI). The antisense probe served as a negative control. Finally, sections were mounted, and a cover slip was applied with neat fluorescent mounting media. Images were acquired via an Leica DM2500 Microscope.

### 2.11 Establishment of myeloid cell specific IL‐6‐deficient Bone Marrow (BM) transplantation model in mice

As described in our previous work,^20^ 4‐ to 10‐week‐old (B6 × 129) F2 (B6.129) mice homozygous for IL‐6 mutation and their littermate controls were donated by Dr. Yu, West China Hospital, Sichuan University. IL‐6^−/−^ or littermate derived BM cells were isolated from their femur, tibia and humerus. Ten‐week‐old recipient Balb/c mice were fed with medicated water 1 week prior to irradiation. To ablation the bone marrow, mice were exposed to a dose of 8 Gy γ‐irradiation. Then the mice were transplanted with 2 × 10^6^ BM cells donated from IL‐6^−/−^ or littermate donors via tail vein within 6 hours following ion exposure. After 4‐week BM reconstitution, mice were intraperitoneally injected with 400 mg/kg BW of acetaminophen for acute liver injury induction.^20^, ^21^ A mouse cytokine/chemokine Magnetic bead panel (MCYTMAG70PMX25BK, Millipore) was used to determine the serum levels of inflammatory factors in mice. All procedures complied with and were approved by the Sichuan University Health Science Institutional Animal Care and Use Committee.

### 2.12 Mouse monocyte isolation

The blood of mice was collected by removing eyeball into EDTA vacutainer tubes, and erythrocytes were lysed using red blood cell (RBC) lysis solution for 5 minutes at room temperature. Cells were centrifuged at 1000 g for 3 minutes to remove RBC lysis solution, and the leucocyte pellet was resuspended and washed with PBS. Leucocyte viability was counted via trypan blue exclusion method. Two‐colour flow cytometry analysis was performed on leucocytes. Unfractionated cells were stained with FITC‐conjugated anti‐CD11b (BD Biosciences) and Alexa Fluor^®^647‐conjugated anti‐CCR2 (Biolegend, San Diego, CA). Cells were incubated at room temperature for 40 minutes, washed with PBS and stored at 4°C until the analysis. At least 50 000 cells were used per experiment.

### 2.13 Statistical analysis

All data are expressed as mean ± SEM. Statistical analysis was performed with Graphpad Prism Software version 5.0 (GraphPad Software Inc., La Jolla, CA). Continuous variables were compared by Student's *t* test or the ANOVA test. If the test result of the variance homogeneity between the groups was significant, the Mann‐Whitney *U* test was appropriately adopted. Non‐parametric statistic was used in samples with non‐normal distribution and/or elevated dispersion. A value of *P* < .05 was considered statistically significant.

## 3 RESULTS

### 3.1 Monkeys challenged by toxins develop severe acute liver failure

Seven adult rhesus monkeys were intraperitoneally administered with toxins (Figure 1A). After a latent period of around 2 days, the monkeys began to show poor appetite, vomiting and torpidity, followed by grasping disability, mental indifference, drowsiness, asterixis and hepatic coma, each of which gradually appeared and worsened. The monkeys were killed when a deep coma developed between day 5 and day 9 (Figure 1A). Because the monkeys became extremely sick and had a high risk of bleeding, the final biopsies were conducted on day 4 with blood sampled on day 5. Hepatic indicators, including alanine aminotransferase (ALT), glutamic‐oxaloacetic transaminase (AST), blood ammonia (BA), bilirubin, clotting time and lactate dehydrogenase (LDH), continued to rise and increased by dozens to hundreds of folds (Figure 1B). The biopsies results showed that the livers developed mild‐to‐moderate steatosis and focal necrosis before day 2 and lethal steatosis, patchy necrosis and sinusoid hyperaemia appeared at later times (Figure 1C). The necropsies revealed that the failed livers exhibited a shrunken appearance and were yellow and red in colour. In addition, histology revealed clear evidence of severe piecemeal necrosis and parenchymal haemorrhage (Figure 1D).

### 3.2 Adaptive immune responses are stable in the development of ALF

**Figure 2:**
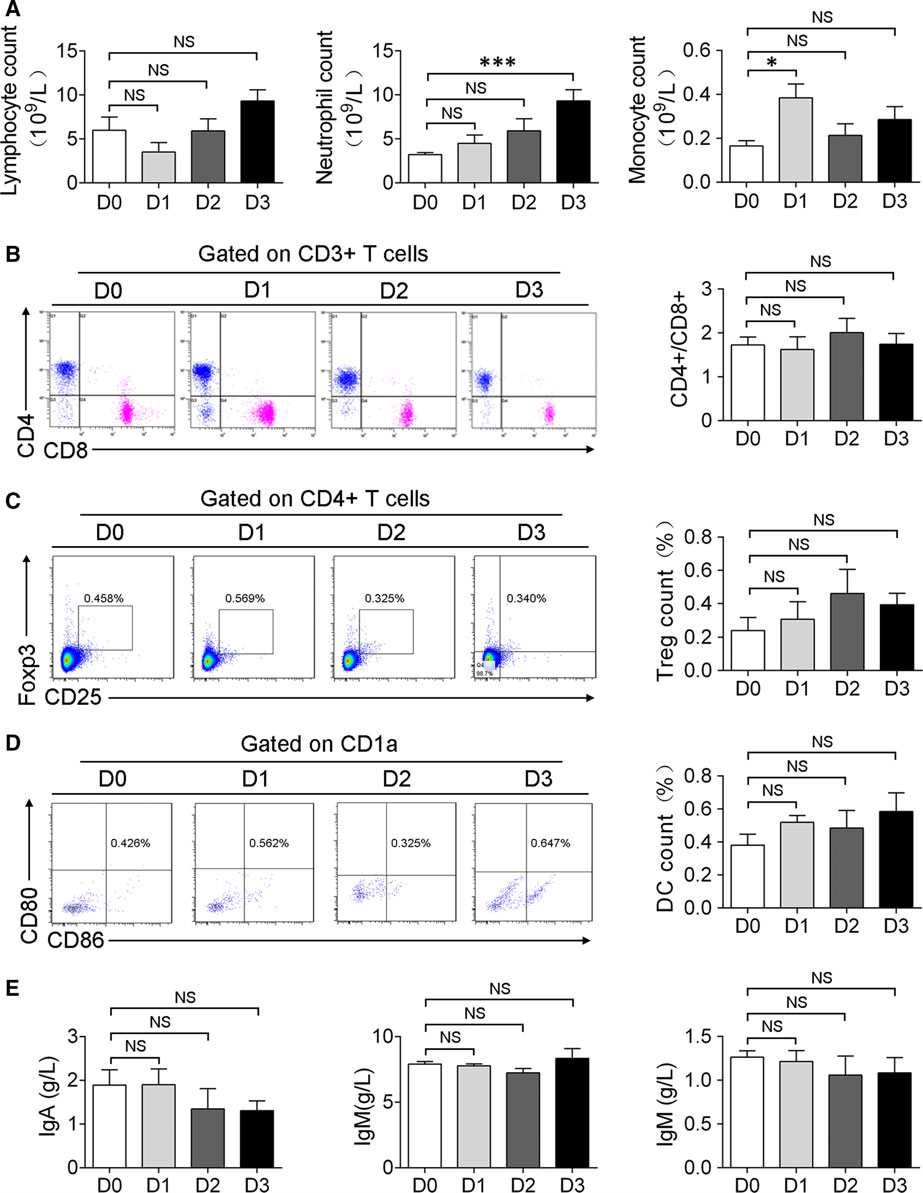
Changes in the circulating inflammatory cells and immunoglobins. A, Changes in the counts of circulating lymphocytes, neutrophils and monocytes. B, Flow cytometric analysis and quantitation of the ratio of CD4+/CD8+ T cells. C, Flow cytometric analysis of CD4+/CD25+FoxP3+ regulatory T cells. D, Flow cytometric analysis of CD1a+/CD80+/CD86+ dendritic cells. E, The change of immunoglobulin. Data represent the means ±SEM; n > 3; NS, not significant. *P < .05, ***P < .001 compared with the data at D0

Although the innate immune system plays a dominant role in liver immunity, in certain drug or pathogen‐induced liver injuries, T cells and B cells can either augment liver damage or regulate hepatic immune responses.^22^, ^23^ We next analysed alterations in the components of the adaptive immune system. Little changes in the numbers of peripheral lymphocytes (Figure 2A), the ratio of CD4+/CD8+ T cells (Figure 2B), the proportion of regulatory T cells (Tregs, CD4+CD25+FoxP3+; Figure 2C) and mature dendritic cells (DCs, CD1a+/CD80+/CD86+; Figure 2D) and the levels of immunoglobulins, including IgM, IgG, and IgA (Figure 2E), were observed during the course of the analysis.

### 3.3 Toxin‐treated monkeys develop severe systemic inflammation

Massive liver injury can cause local and systemic inflammatory responses that lead to multi‐organ failure and death.^7^, ^11^ Although neutrophil‐mediated acute liver injuries have been reported in some cases of sterile inflammation,^24^ we found that the neutrophil counts in the toxin‐treated monkeys remained stable or increased slightly (Figure 2A). In addition, we did not observe clear hepatic aggregation of polymorphonuclear cells, even in the failed livers (Figure 1D,E).

**Figure 3:**
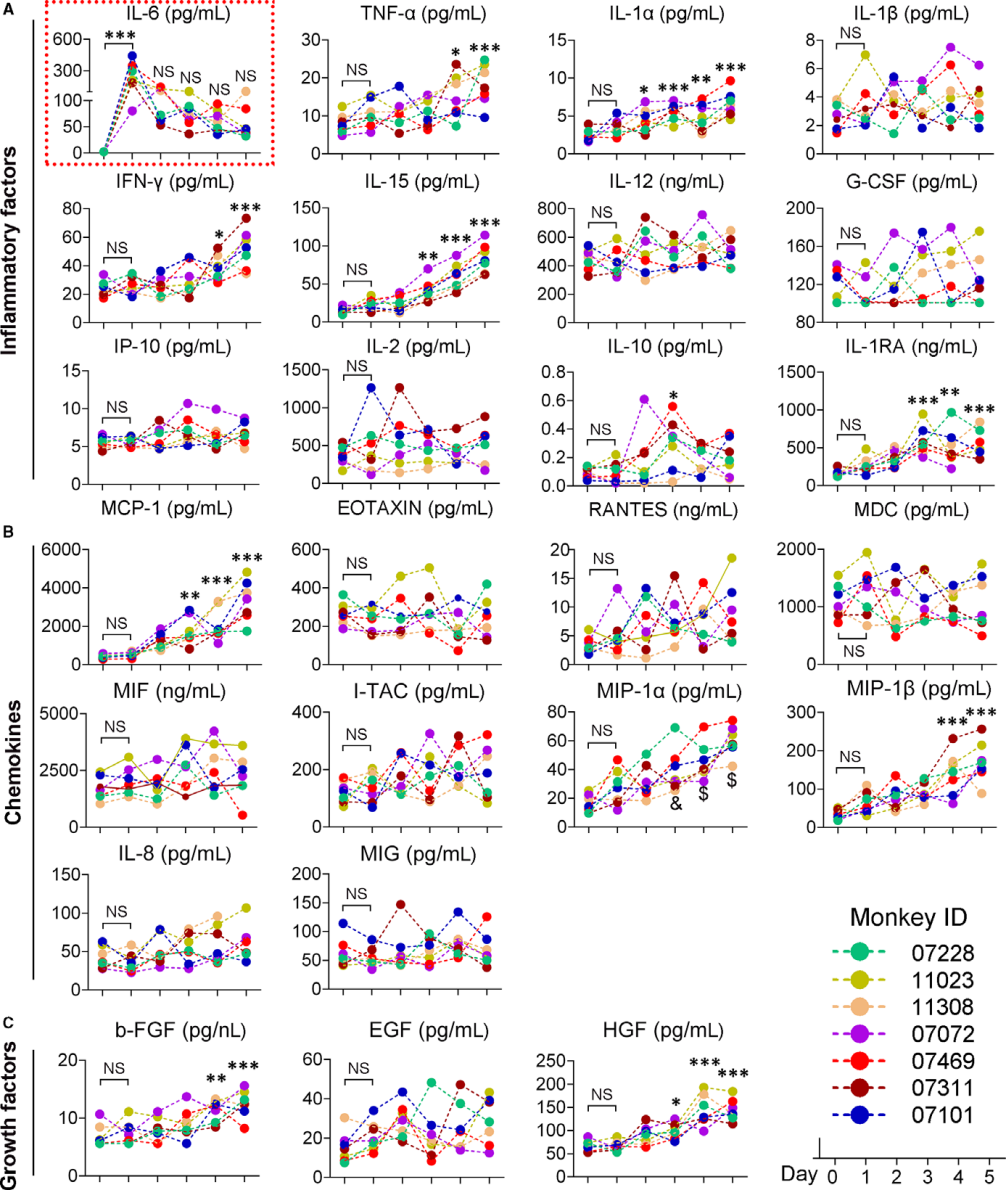
Serum levels of inflammatory cytokines, chemokines and growth factors. (EGF, epidermal growth factor; Eotaxin, eosinophil chemotactic factor; FGF, fibroblast growth factor; G‐CSF, granulocyte colony‐stimulating factor; HGF, hepatocyte growth factor; IL, interleukin; INF‐γ, interferon γ; IP‐10, interferon‐inducible protein‐10; I‐TAC, interferon‐inducible T‐cell α chemoattractant; MCP‐1, monocyte chemoattractant protein‐1; MDC, macrophage‐derived chemokine; MIF, macrophage migration inhibitory factor; MIG, monokine induced by interferon‐γ; MIP‐1α, macrophage inflammatory protein‐1α; Rantes, regulated upon activation normal T‐cell expressed and secreted; TNF‐α, tumor necrosis factor). NS, not significant; *P < .05, **P < .01, ***P < .001 compared with the data at D0

We then assessed the levels of circulating inflammatory factors. The levels of most factors, including the pro‐inflammatory cytokines TNF‐α, IL‐1β, IL‐1RA, IL‐15 and IFN‐γ, the chemokines MCP‐1, MIP‐1α and MIP‐1β, and the growth factors HGF, VEGF and FGFb, did not exhibit any statistical increase within the first day but began to steadily increase on day 2 and reached their peak levels before the animals were sacrificed (Figure 3). Other factors, including IL‐2, IL‐8, IL‐10, IL‐12, G‐CSF, EGF, Rantes, Eotaxin, MIF, I‐TAC, MDC, MIG and IP‐10, also increased to varying degrees (Figure 3). Most strikingly, different to other factors which began to significantly increase at day 2, we observed a 30‐ to 160‐fold increase in IL‐6 as early as 1 day after toxin injection. IL‐6 levels then dropped precipitously on day 2 but remained very high (Figure 3).

### 3.4 IL‐6 is principally produced by circulating monocytes

**Figure 4:**
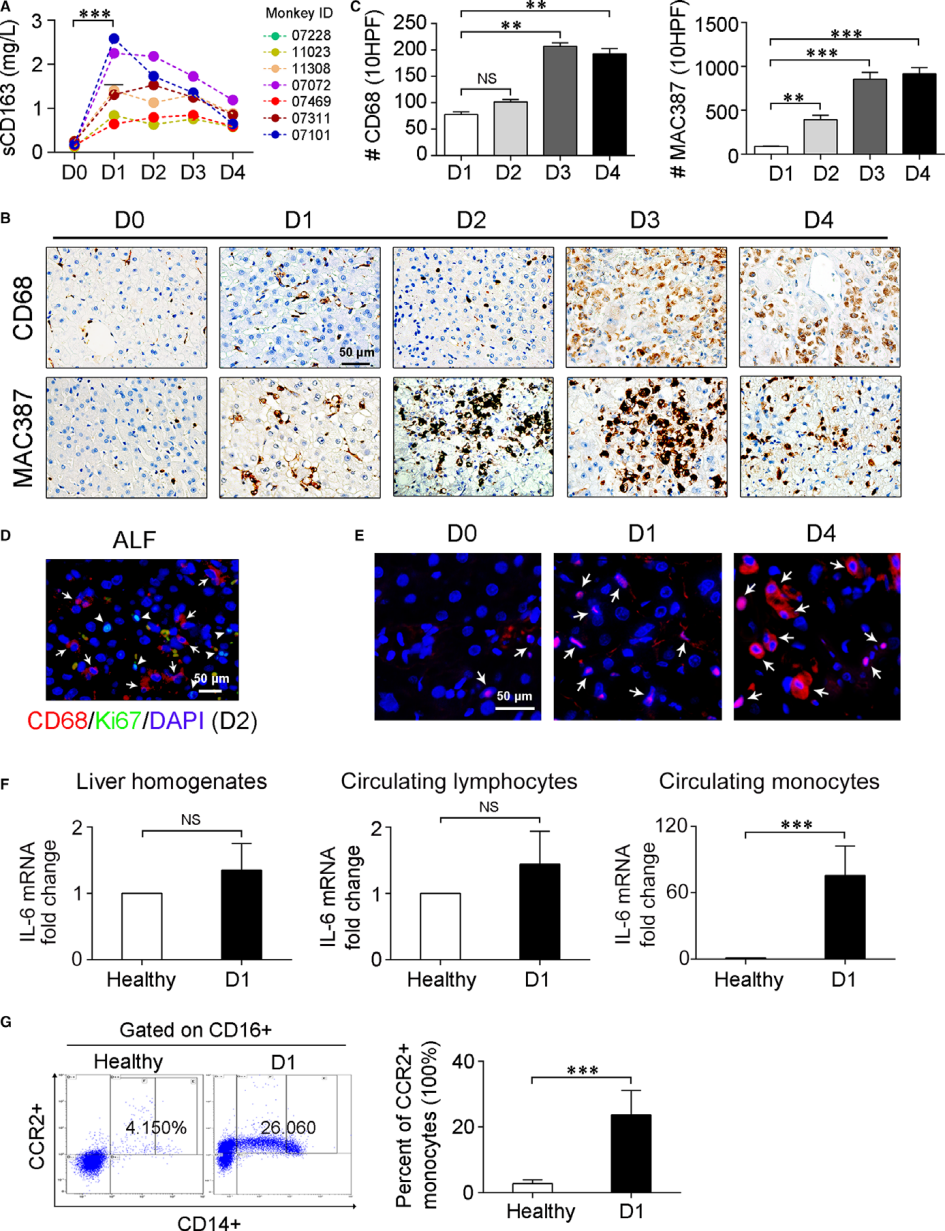
Activation of circulating monocytes in monkeys with acute liver failure. A, ELISA measurement of serum levels of sCD163 in the treated monkeys at indicated times; n = 7; ***P < .001. B, Representative photomicrographs of the results of immunohistochemical staining to identify resident KCs (CD68+) and recruited hepatic macrophages (MAC387+) in liver tissues. C, Quantitation of CD68+ and MAC387+ macrophages. The numbers of CD68+ and MAC387+ cells were counted in 10 consecutive high‐power fields in 3 monkeys. For each section, 10 randomly chosen portal tracts or areas of central veins were assessed at high magnification (×400), and the cumulative number of positive cells in 10 HPF was recorded. Data represent the means ± SEM; n = 7; **P < .01, ***P < .001. D, Double immunofluorescence staining for CD68 and Ki67 in liver tissues. Note that the Ki67‐positive cells (indicated by arrowheads) are hepatocytes but not CD68+ KCs (arrows). E, FISH assay for IL‐6 mRNA in biopsy liver tissues at different time points. Macrophages are indicated by arrows identified by their shape, size and lobular location. F, Transcript levels of IL‐6 in liver homogenates, circulating monocytes, and lymphocytes. Each bar represents data obtained from at least 3 independent quantitative PCR experiments. Data represent the means ± SEM; n > 3; NS, not significant. ***P < .001. G, Flow cytometric analysis of activated monocytes (CD14+CD16+CCR2+) in subsets of peripheral blood monocyte. Data represent the means ± SEM; n = 7; ***P < .001

Synthesis of IL‐6 by various cells has been reported. Hepatocytes even can produce IL6 under some common hepatic stimuli.^25^ However, in injured livers, KCs are regarded as the principal producers.^26^, ^27^, ^28^ The rapid and sharp increase in IL‐6 in the monkeys suggested that a massive activation of KCs had occurred. In addition, the levels of soluble CD163 (sCD163), which is released uniquely by activated macrophages,^29^ increased robustly after 1 day (Figure 4A), providing further evidence for the immediate activation of macrophages.

We then stained the liver specimens for CD68, a marker of activated KCs.^30^ Unexpectedly, we found that although CD68 staining increased substantially with the progression of ALF, no noticeable aggregation of CD68+ cells was observed before day 2 (Figure 4B,C). In adults, the expansion of hepatic macrophages can occur either from the self‐renewing resident KCs or from recruited c‐Mos.^10^, ^29^, ^30^ We rarely observed proliferating KCs that were double‐labelled with CD68 and Ki67 (Figure 4D). In contrast, c‐Mos‐derived macrophages positive for MAC387^10^, ^30^ were extensively localized in the parenchyma at day 2 (Figure 4B,C).

IL‐6 appeared to be produced primarily by extrahepatic cells, especially during the early stage after injury. This was further supported by evidence showing that the levels of IL‐6 mRNA in hepatic homogenate increased slightly at day 1 and very weak fluorescent signal was probed in the KCs by fluorescent in situ hybridization assay (Figure 4E). With the hepatic aggregation of c‐Mos at day 4, the IL‐6 mRNA was remarkably increased (Figure 4E). After excluding the possibility that circulating lymphocytes contributed to the elevation of IL‐6 levels (Figure 4F), we investigated whether c‐Mos secreted IL‐6 before undergoing hepatic migration. Indeed, at day 1, the total number of c‐Mos was significantly increased (Figure 2A); moreover, the monocytes isolated from the toxin‐treated monkeys expressed much higher levels of IL‐6 mRNA (Figure 4F). Notably, the proportion of activated CD14+CD16+CCR2+ monocytes^6^, ^31^ increased substantially in these animals (Figure 4G).

### 3.5 Monocyte‐derived IL‐6 is critical for augmenting liver damage and homeostatic disarrangement in mouse

**Figure 5:**
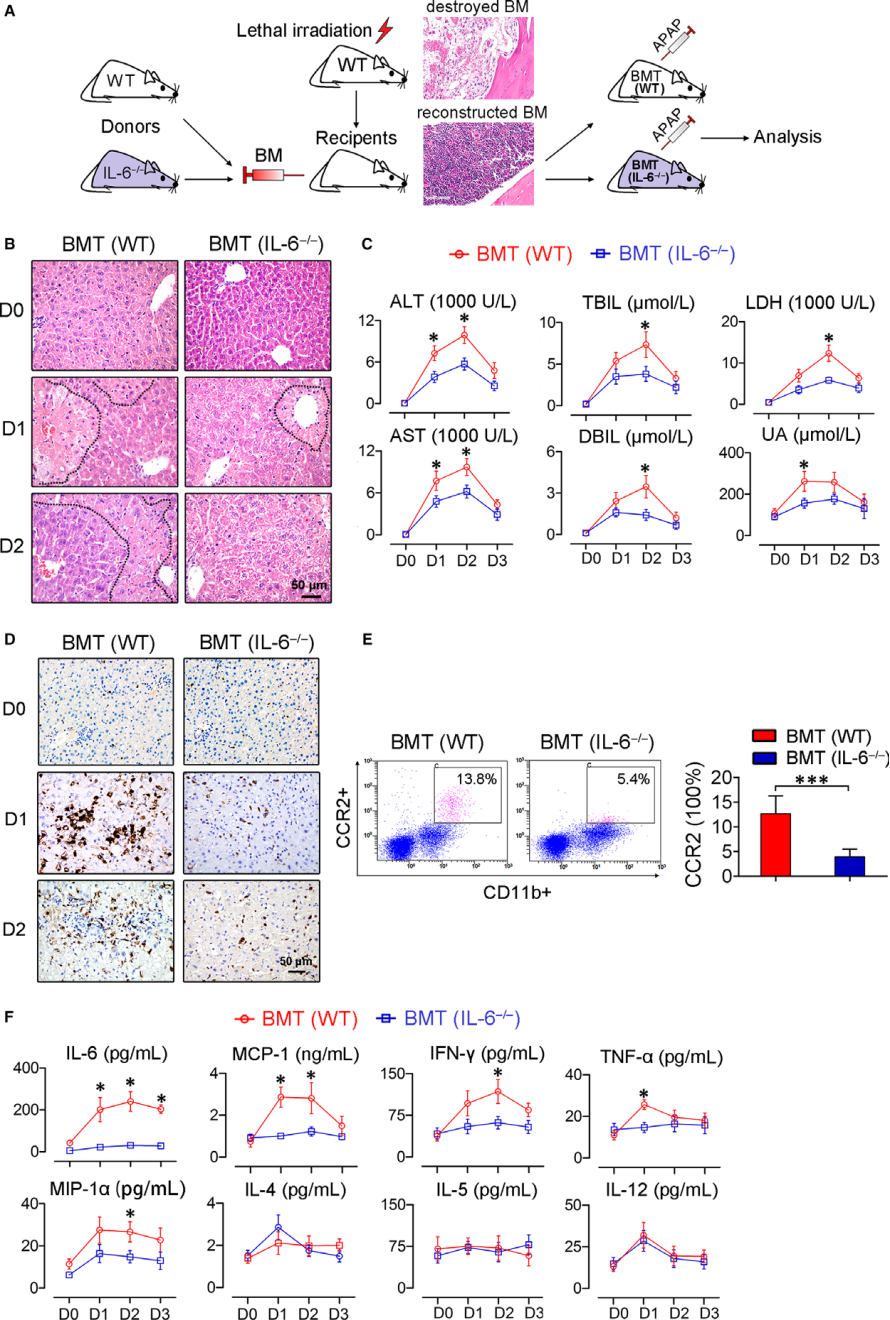
Depleting IL‐6 in myeloid cells decreases toxin‐induced liver damage in mice. A, Schematic representation of the experimental design. The inserted H&E stained tissue sections show the deconstructed (upper) and reconstructed bone marrow (lower). B, H&E staining of the liver tissue shows improved hepatic histology was observed in the mice with IL‐6‐deficient bone marrow. The necrotic zones are circled with black dotted lines. C, Biochemical assays of hepatic indexes; n = 6. D, Macrophage aggregation in the liver as indicated by immunohistochemical staining for F4/80. E, Flow cytometric analysis of the activated monocytes (CD11b+CD16+CCR2+) in the peripheral blood monocyte subsets; n = 4. F, Circulating factors were suppressed in the mice that received IL‐6‐deficient bone marrow after toxin challenge; n = 6. All the data represent the means ± SEM; *P < .05, ***P < .001

The drastic increase in IL‐6 occurred much earlier than other factors, suggesting that IL‐6 is a critical trigger that exacerbates systemic inflammation. Because c‐Mos were the major source of IL‐6 in these animals, we next explored the role of monocyte‐derived IL‐6 in these pathophysiological processes. We established a mouse model, in which we first ablated the bone marrow (BM) using ionic radiation and then reconstituted it by infusing BM from an IL‐6‐deficient mouse (Figure 5A). This model was used because (i) systemically depleting IL‐6 can result in spontaneous liver damage and impaired liver regeneration^32^ and (ii) a monocyte‐specific IL‐6‐deficient mice were lacking. Once the BM was successfully reconstituted, the IL‐6 gene was selectively ablated from nucleated myeloid cells, including granulocytes, DCs, monocytes and macrophages. Because granulocytes and DCs rarely produce IL‐6,^28^ the mice could be regarded as IL‐6‐deficiency‐specific for monocytes. When they were challenged with intraperitoneally injected acetaminophen, a well known hepatotoxic substance,^13^, ^31^ the mice that received IL‐6‐deficient BM exhibited much less liver damage (Figure 5B,C), lower levels of c‐Mos activation (Figure 5D,E) and significantly lowered local and systemic inflammatory responses (Figure 5F) than the those receiving BM from wild‐type donors.

## 4 DISCUSSION

Because the pathogenesis of ALF is not fully understood, no efficient therapeutics have been developed to treat this condition.^1^, ^2^, ^3^, ^4^, ^5^, ^14^ In the present study, we used a large non‐human primate model to panoramically and dynamically reveal the cellular and molecular events that lead to ALF. We found that c‐Mos and their product, IL‐6, play critical roles in initiating and accelerating ALF.

The large species difference between small animals and human, as well as the unavailable early information of the changes in liver tissue and internal environment, have extremely hindered the understanding of the pathophysiological processes in ALF.^13^, ^15^ We have previously established a non‐human primate model of toxin‐induced ALF which displayed similar changes in clinical features, hepatic indexes, histopathology, imaging and lifespan with ALF patients in the clinic.^17^ In the present study, we used this non‐human primate model of ALF to investigate the mechanisms underlying the initiation and development of ALF.

It has been reported that amanitin could be completely removed within 24 hours^18^; however, it is worth noting that during the first 2 days after the toxins were injected, we observed no significant differences in the hepatic parameters. In addition, the liver did not exhibit any noticeable histological injuries. Therefore, the degrees of primary toxic hepatic impairment appeared to be much less severe than what we have previously imaged, which highlighted the role of secondary systemic inflammation in promoting this fatal condition. It is well documented that components of innate immunity, especially resident KCs play central roles in promoting ALF.^10^, ^30^ However, the precise molecular and cellular events that lead to ALF remain obscure. As the most immediately and dramatically increased cytokine in this disease, IL‐6 appears to play a central role in exacerbating the “cytokine storm”.^33^, ^34^ By acting as a pro‐inflammatory cytokine, IL‐6 targets numerous genes that are known to regulate liver repair following a diverse array of injuries. However, overproducing IL‐6 often leads to excessive inflammatory reaction, the exacerbation of tissue damage and, occasionally, aberrant liver regeneration and tumorigenesis.^28^, ^35^ Although IL‐6 is mainly produced by macrophages and lymphocytes, we did not observe that lymphocytes showed any elevation in IL‐6 producing. It has been reported that in response to a diversity of hepatic injuries, IL‐6 is principally produced by KCs, which make up over 80% of the total fixed macrophages in the body.^27^, ^28^ Unexpectedly, we did not find that there was a noticeable increasing in the number of KCs and the transcription of IL‐6 mRNA. We observed that c‐Mos were the major source of IL‐6 before they were recruited to the liver and differentiated into mature macrophages. Moreover, ablating IL‐6 in mouse myeloid cells resulted in extremely limited c‐Mos activation, suggesting that c‐Mos are capable of stimulating itself by releasing IL‐6.

**Figure 6:**
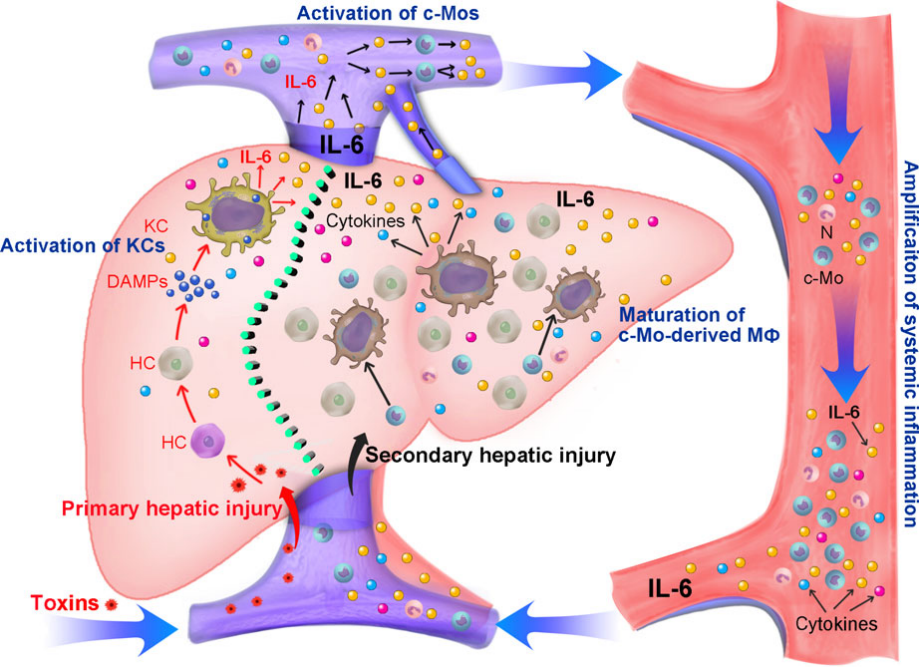
A proposed cellular and molecular model of the mechanisms underlying acute liver failure. (c‐Mos, circulating monocytes; DAMPs, damage‐associated molecular patterns; HC, hepatocytes; KCs, Kupffer cells; MΦ, macrophages; N, neutrophill)

It would be interesting to identify the “first signal” that fuels c‐Mos activation. A reasonable possibility is that, as illustrated in Figure 6, upon stimulation by pathogens or cell debris, resident KCs release the earliest alarm signal, most likely IL‐6, to recruit more c‐Mos. The activated c‐Mos then release more IL‐6 to activate more c‐Mos. As a result, there is an explosive increase in the number of activated c‐Mos and a dramatic increase in the secretion of IL‐6. These events subsequently activate a secondary “cytokine storm,” which involves the unavoidable activation of effector cells and the worsening of homeostasis. Our results suggest that disrupting the cytokine cascade could be a potentially viable therapeutic approach for treating ALF and that c‐Mos might be a promising target for such therapeutic strategies.

In summary, we used a large, non‐human primate model to provide novel insights into the cellular and molecular events underlying the initiation and acceleration of ALF. Our work demonstrates that circulating monocytes play a critical role in the augmentation of systemic inflammation before their hepatic aggregation and mature differentiation, acting as a potential therapeutic target for this lethal syndrome, and our findings also encourage early therapies that against the activation of c‐Mos prior to the full development of a “cytokine storm.”

## CONFLICT OF INTEREST

Nothing to report.
