#  

**DOI:** 10.1111/jcmm.17050

**Published:** 2021-12-03

In Gang Guo,^1^ the published version contains an error in the bottom left image in Figure 5D. The correct figure is shown below. All results and conclusions remain intact. The authors apologize for the errors.

**FIGURE 5:**
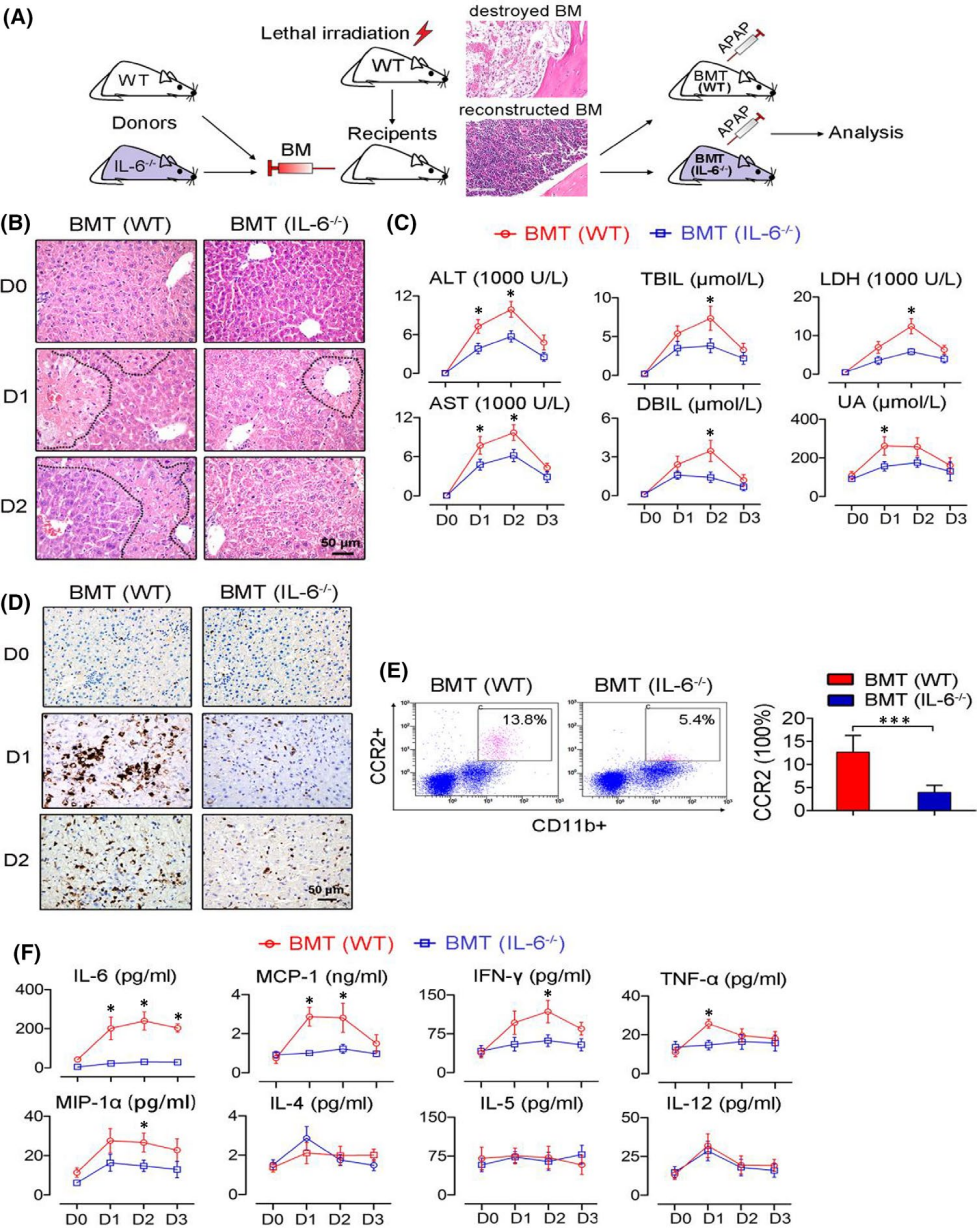
Depleting IL‐6 in myeloid cells decreases toxin‐induced liver damage in mice. (A) Schematic representation of the experimental design. The inserted H&E‐stained tissue sections show the deconstructed (upper) and reconstructed bone marrow (lower). (B) H&E staining of the liver tissue shows improved hepatic histology was observed in the mice with IL‐6–deficient bone marrow. The necrotic zones are circled with black dotted lines. (C) Biochemical assays of hepatic indexes; *n* = 6. (D) Macrophage aggregation in the liver as indicated by immunohistochemical staining for F4/80. (E) Flow cytometric analysis of the activated monocytes (CD11b^+^CD16^+^CCR2^+^) in the peripheral blood monocyte subsets; *n* = 4. (F) Circulating factors were suppressed in the mice that received IL‐6–deficient bone marrow after toxin challenge; *n* = 6. All the data represent the means ± SEM; *P < 0.05, ***P < 0.001
